# Histology and morphometry of the skin of the Korean ice goby *Leucopsarion petersii* (Gobiiformes, Gobiidae), in relation to its ecology and habitat

**DOI:** 10.1186/s42649-026-00136-8

**Published:** 2026-05-11

**Authors:** Mu Sung Sung, Hyun Tae Kim

**Affiliations:** 1 Department of Convergence Environmental Science, Dongguk University, 32, Dongguk-ro, Ilsandong-gu, Goyang-si, Gyeonggi-do 10326 Republic of Korea; 2 Jeonju National University of Education, Jeonju, 560~757 Republic of Korea

**Keywords:** Epidermal thickness, Basement membrane thickness, Mucous cell, Club cell, Skin histology, *Leucopsarion petersii*, Morphometric variation

## Abstract

The histological structure and morphometric characteristics of the skin of the Korean ice goby *Leucopsarion petersii* were investigated using light microscopy, two histological staining techniques, and SPSS statistical analysis. Skin samples from adult *L*. *petersii* fishes were collected from five body regions, including the head, operculum, dorsal body, lateral body, and ventral body. In all examined regions, the skin of *L*. *petersii* exhibited the general structure as in other teleost fishes, consisting of an epidermis and dermis separated by a distinct basement membrane with underlying skeletal muscle. The epidermis was composed of outermost flattened cells, basal cells, club cells, and mucous cells. However, some histological findings differed from the typical teleost fishes: I), the dermis was mainly characterized by a well-developed stratum compactum with an almost complete absence of the stratum laxum; II), Although epithelial thickness showed significant regional variation, it was positively correlated with basement membrane thickness (*r* = 0.417, *p* < 0.001; *n* = 100); III), Mucous cells, suggesting the formation of a protective mucosal barrier on the skin surface, were distributed in the outer epidermal layer; IV), club cells, implying retention of chemical defense mechanisms against environmental stress, were confirmed within the epidermis. Consequently, the skin histology of the skin of *L. petersii* may reflect adaptive responses to its thin body surface and to the fluctuating environmental conditions encountered in coastal and estuarine habitats.

## Introduction

The skin of teleost fishes represents the sole boundary between the external aquatic environment and internal tissues (Cabillon and Lazado 2019), and is therefore recognized as a critical organ system that determines their survival rather than serving merely as a protective layer (Reyes-López et al. 2021). Aquatic environments are characterized by constant exposure to pathogens, suspended particulates, and ongoing physical abrasion (Benhamed et al. 2014). As a result, the skin and the mucus secreted from it generally function as the first line of defense, blocking external threats at the earliest point of contact (Dash et al. 2018). Moreover, the mucus layer contains antimicrobial and immune-related compounds, providing not only physical but also chemical and immunological defense functions (Ángeles Esteban 2012). In addition to these defensive roles, the skin is broadly involved in sensory reception, coloration, and intra- and inter-specific signaling (Reyes-López et al. 2021). In fishes, therefore, the skin is a complex tissue system that integrates defense, homeostasis, environmental sensing, and ecological interaction functions (Reyes-López et al. 2021). The variety of functional demands and their manifestation differ among species, being concretized by differences in skin thickness, epithelial cell composition, mucus secretion patterns, and microstructural traits (Sire and Akimenko 2004). Accordingly, the morphological and functional characteristics of fish skin are determined by species-specific habitat conditions and life-history strategies, and analyses of cutaneous form and function therefore provide critical insights into the mechanisms by which fishes achieve ecological adaptation to their environments (Gomez et al. 2013).

The Korean ice goby *Leucopsarion petersii* is a small species distributed in the northwestern Pacific, including the coastal waters and estuary of Korea, Japan, and China, and exhibits an anadromous migratory behavior in which individuals grow in marine environments and migrate into estuarine and brackish waters for spawning (Kim and Park 2002). The ice goby completes a relatively short life cycle within transitional environments characterized by pronounced spatio-temporal fluctuations in salinity and hydrodynamic conditions, during which it is continuously exposed to abrupt changes in osmotic pressure and water flow (Evans et al. 2005). Owing to this distinctive life history, the ice goby can be regarded as occupying a unique ecological position that requires simultaneous physiological and morphological adaptations. In particular, the ice goby possesses an extremely thin and transparent external morphology, which represents a taxonomically important trait distinguishing it from other gobiid fishes (Goda and Fujii 1996) and directly suggests the presence of specialized skin structures. Despite these distinctive life-history and unusual appearance, virtually no studies have examined the morphological and structural strategies of the skin that enable the ice goby to survive and adapt to rapid environmental changes. Therefore, this study aims to comprehensively investigate the adaptive properties of the skin of the ice goby, with special emphasis on changes associated with its migration from marine environments to brackish spawning grounds, and to provide fundamental information on skin-based adaptive strategies in small migratory fishes inhabiting transitional waters.

## Materials and methods

### Specimen collection and fixation

**Fig. 1:**
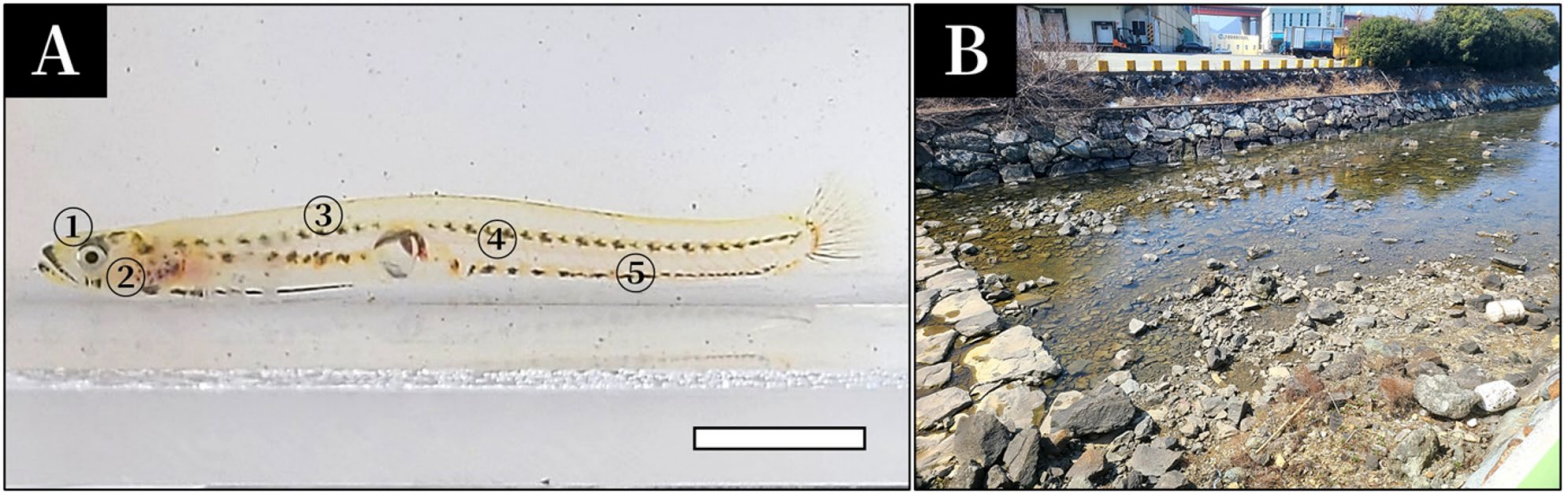
Photograph (**A**) and habitat (**B**) of the ice goby *Leucopsarion petersi*i. **A**, the five body regions analyzed for skin histology: head (①), operculum (②), dorsal body (③), lateral body (④), and ventral body (⑤). The bar indicates 1 cm

In April 2026, a total of 15 adult ice gobies (Fig. 1A) were collected from an estuarine area in Sambong-ri, Samsan-myeon, Goseong-gun, Gyeongsangnam-do, South Korea (Fig. 1B). Captured individuals were immediately transferred to indoor tank in the laboratory and anesthetized using tricaine methanesulfonate (MS-222; Sigma-Aldrich, USA). For histological observations, specimens were fixed in 10% neutral buffered formalin (pH 7.4). All experimental and handling procedures were conducted in accordance with the ethical guidelines and regulations of the Institutional Animal Care and Use Committee of Jeonbuk National University.

## Microscopy Procedures

After fixation, skin tissues were carefully excised from each skin region using fine surgical instruments: top of head, operculum, dorsal body, lateral body, and ventral body (Fig. 1A). For light microscopy, formalin-fixed samples were thoroughly rinsed in running tap water, dehydrated through a graded ethanol series, cleared in xylene, and embedded in paraffin wax. Paraffin blocks were sectioned at a thickness of 5 μm using a rotary microtome. The resulting serial sections were stained with hematoxylin and eosin (H&E) for general tissue structure and with Masson’s trichrome to visualize connective tissue components. Stained sections were examined and photographed using a light microscope (Carl Zeiss, Oberkochen, Germany).

### Statistical analysis

All statistical analyses of cutaneous morphometric parameters in *L. petersii* were conducted using IBM SPSS Statistics software (version 18.0; IBM Corp., USA). Epidermal thickness was evaluated for each skin region and was measured as the linear distance from the basement membrane to the apical surface of the epidermis. In addition, basement membrane thickness was measured as the perpendicular distance across the basement membrane at the same sampling sites. A total of four individuals were selected, and measurements were taken at five locations for each individual. Prior to inter-regional comparisons, normality of the data was assessed using the Shapiro–Wilk test, and homogeneity of variances was evaluated using Levene’s test. When the assumptions required for parametric analysis were satisfied, one-way analysis of variance (ANOVA) was performed to compare regional means. When the assumption of normality was violated despite homogeneity of variances being satisfied, differences among groups were analyzed using the Kruskal–Wallis test. Post hoc comparisons were conducted using Scheffe’s test. The significance level for all statistical tests was set at *p* < 0.05. Pearson’s correlation analysis was conducted to evaluate the linear relationship between above two morphometric parameters.

## Results

### Histology

**Fig. 2:**
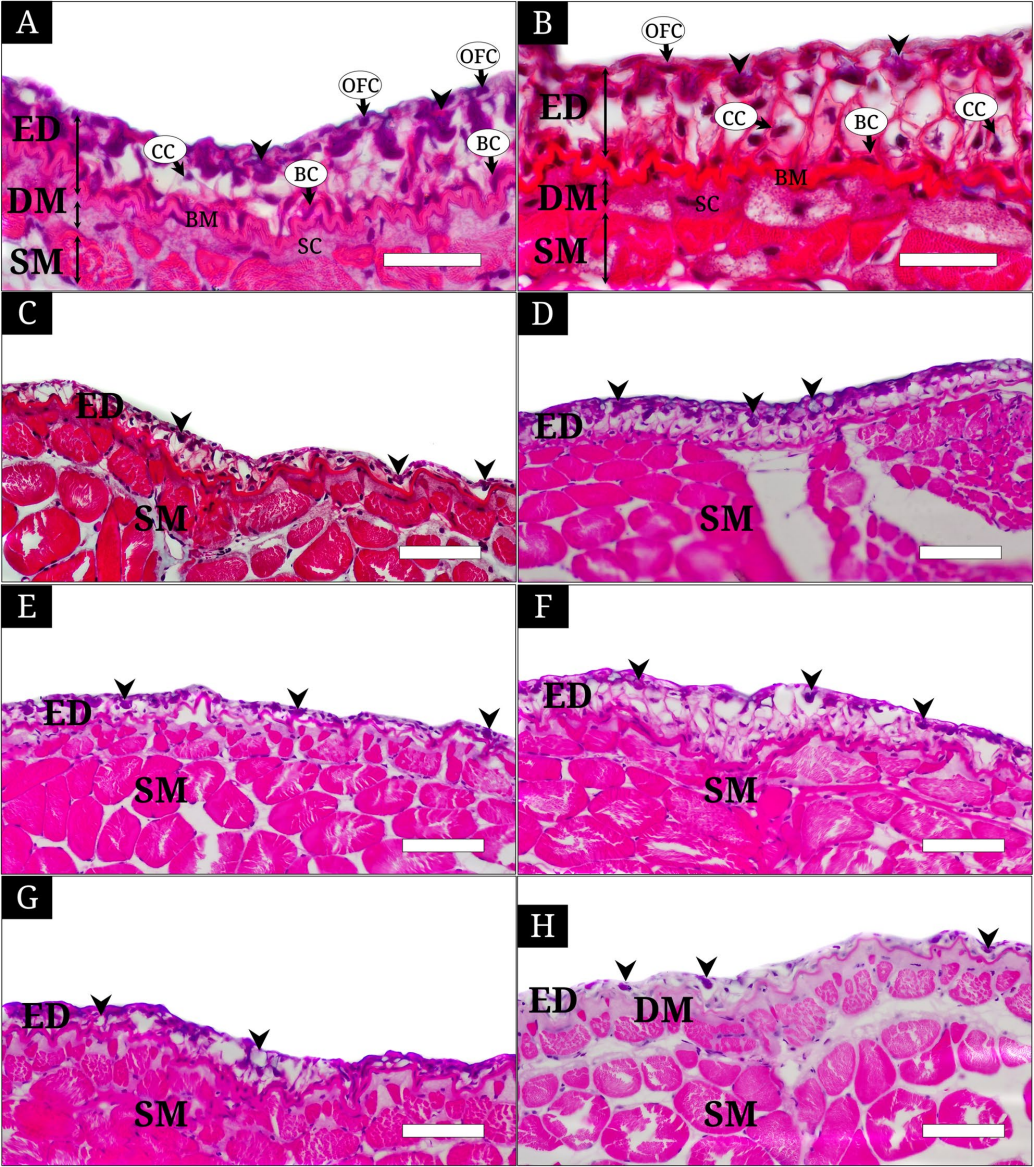
Histological characteristics of the skin of Leucopsarion petersii, stained with hematoxylin and eosin (H&E) (**A**, **C**, **E**–**H**) and Masson’s trichrome (**B** and **D**). **A** and **B**, the head region showing the epidermis (ED) composed of the outermost flattened cells (OFC), the middle layer containing club cells (CC), basal cells (BC), mucous cells (arrowhead) and the dermis (DM) with stratum compactum (SC) separated by a distinct basement membrane (BM), and the underlying subcutaneous muscle (SM); (**C** and **D**), the head region stained with hematoxylin and eosin and Masson’s trichrome; (**E**), the operculum; (**F**), the dorsal body; (**G**), the lateral body; (**H**), the ventral body, respectively, showing regional variation in epidermal thickness and dermal development. The bars indicates 100㎛ in A and B, 200㎛ in C-H

The skin of *L. petersii* was composed of the epidermis (ED) and the dermis (DM), demarcated by a distinct basement membrane (BM), with an underlying skeletal muscle layer (SM) located in the deepest region (Fig. 2). The ED consisted of outermost flattened cells (OFC), basal cells (BC), club cells (CC), and mucous cells (MC). OFCs were situated at the surface layer of the ED, and exhibited flattened to oval nuclei and cytoplasmic morphology. In both H&E and Masson’s trichrome staining, their nuclei appeared purple whereas the cytoplasm was relatively translucent. BCs were located in the deepest portion of the ED, immediately above the BM. These cells possessed flattened or oval nuclei that stained purple in both H&E and Masson’s trichrome preparations, with relatively clear cytoplasm. CCs were observed in the middle layer of the ED and contained relatively small, oval to round nuclei positioned centrally within the cell. In both staining methods, the nuclei appeared purple and were comparatively smaller than those of other epidermal cell types. MCs were distributed mainly in the upper epidermal layer. These cells maintained their structural integrity while producing and secreting mucus to the external surface. The mucus exhibited a purple coloration in H&E staining and a reddish-purple appearance in Masson’s trichrome staining (Fig. 2A and B).

The BM underlying the ED was clearly distinguishable in both H&E and Masson’s trichrome sections, appearing pink and red, respectively, and displayed a markedly undulating morphology. Within the DM, it was identified that an only stratum compactum (SC) exhibited a weak pink coloration in both H&E and Masson’s trichrome staining (Fig. 2A and B).

### Morphometry

**Fig. 3:**
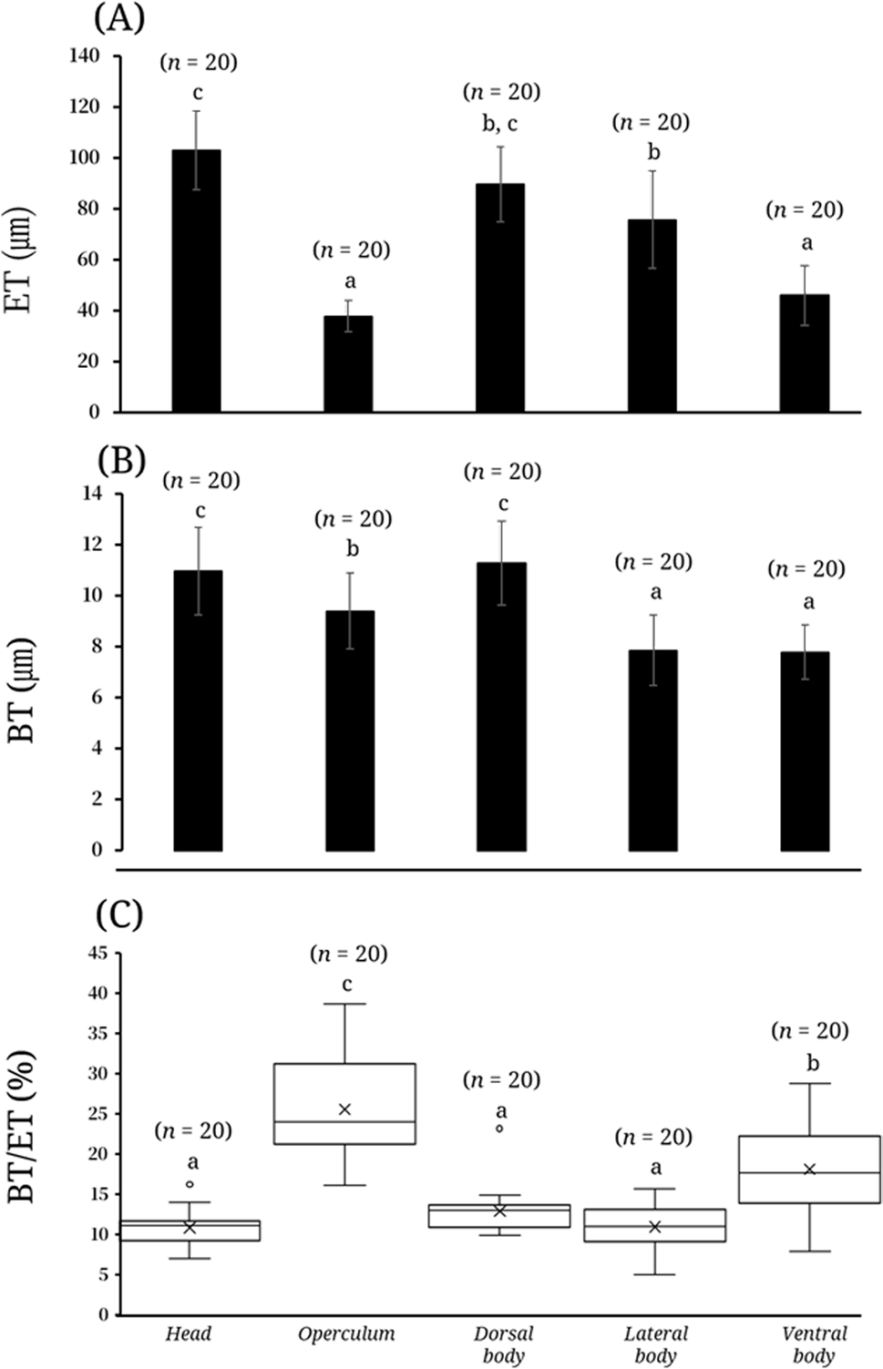
Regional comparison of epithelial thickness (ET), basement membrane thickness (BT), and their relative ratio (BT/ET %) in the skin of Leucopsarion petersii. **A**, mean epithelial thickness among five body regions (head, operculum, dorsal body, lateral body, and ventral body; (**B**), mean basement membrane thickness among the same regions; (**C**), the relative ratio of basement membrane thickness to epithelial thickness. Values are expressed as mean ± SD (n = 20). Different letters above the bars indicate significant differences among regions (p < 0.05)

**Fig. 4:**
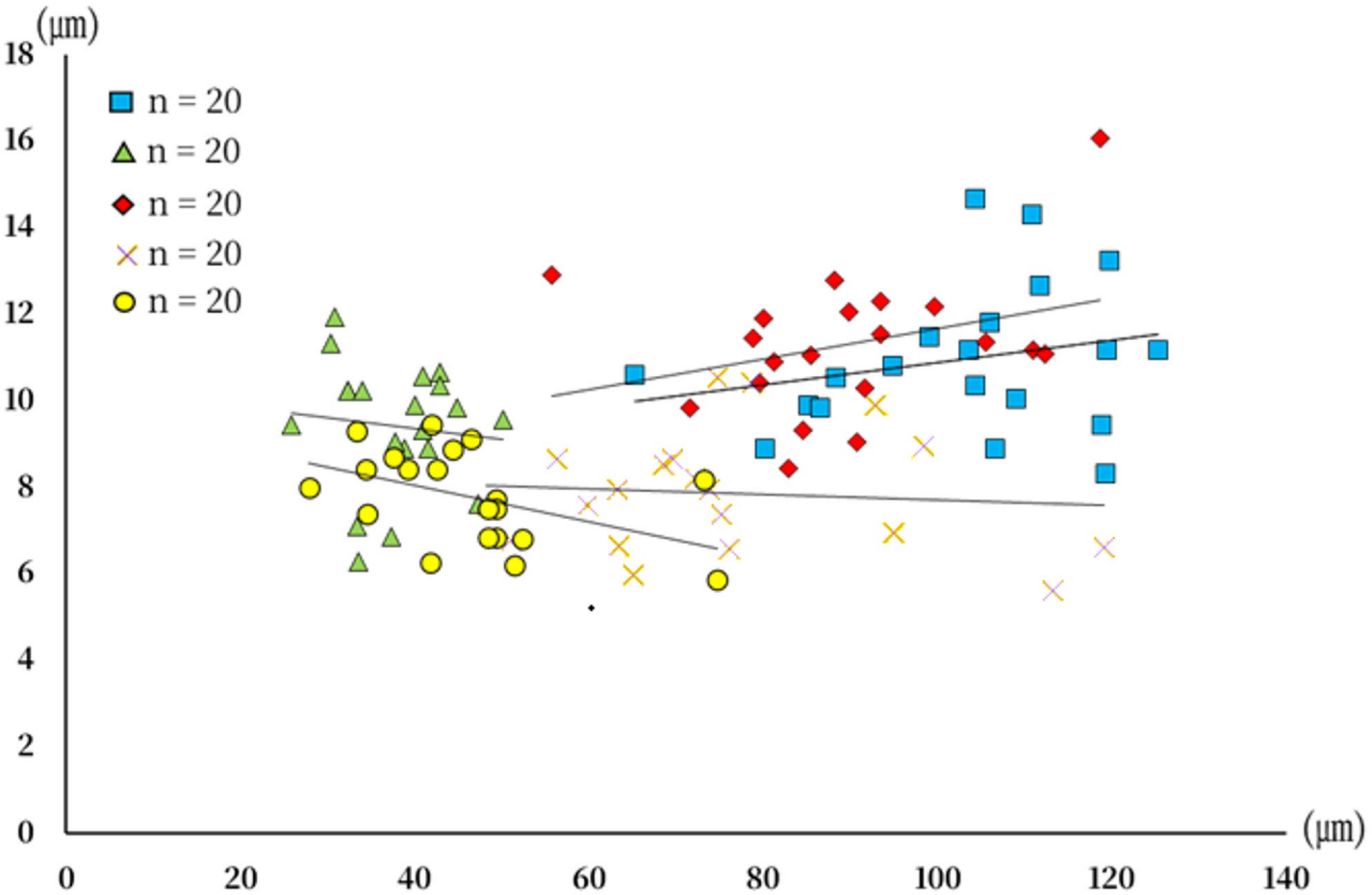
Scatterplot illustrating the relationship between epithelial thickness (x-axis) and basement membrane thickness (y-axis) in five skin regions of L. petersii (n = 20 per region). Blue rectangle, head; green triangle, operculum; red lozenge, dorsal body; x, lateral body; yellow circle, ventral body. Solid lines indicate the linear regression for each region

Measurement of epithelial thickness (ET) revealed clear regional differences. The head exhibited the greatest thickness (mean ± SD = 102.9 ± 15.5 ㎛; range = 65.3–125.2), followed by the dorsal body (89.7 ± 14.8㎛; 55.7–118.6) and lateral body (75.7 ± 19.2㎛; 48.1–119.1). The ventral body showed relatively thinner values (46.0 ± 11. ㎛7; 27.8–74.7), whereas the operculum was the thinnest region (37.9 ± 6.3㎛; 25.9–50.1). These measurements revealed a highly significant difference in ET among regions (Kruskal–Wallis test, χ² = 76.273, df = 4, *p* < 0.001, *n* = 100 measurements from five sites; Fig. 3A). The basement membrane thickness (BT) also differed among the five regions. The dorsal body exhibited the greatest thickness (11.3 ± 1.7㎛; range 8.4–16.1), followed by the head (11.0 ± 1.7㎛; 8.3–14.7) and operculum (9.4 ± 1.5㎛; 6.3–11.9), whereas the lateral body (7.9 ± 1.4㎛; 5.6–10.5) and ventral body (7.8 ± 1.1㎛; 5.9–9.4) showed comparatively thinner values. These results showed a highly significant difference in BT among regions (one-way ANOVA, F = 25.036, df = 4, 95, *p* < 0.001, *n* = 100 measurements from five sites; Fig. 3B). The relative BT to ET % varied among the five regions. The operculum exhibited the highest proportion (25.6 ± 6.6%; range 16.1–38.7), followed by the ventral body (18.1 ± 5.7%; 7.8–28.8) and dorsal body (12.9 ± 2.9%; 9.9–23.2), whereas the lateral body (11.0 ± 2.9%; 4.9–15.7) and head (10.9 ± 2.1%; 7.0–16.2) showed comparatively lower ratios. The relative BT to ET differed significantly among regions (Kruskal–Wallis test, χ² = 59.602, df = 4, *p* < 0.001, *n* = 100 measurements from five sites; Fig. 3C). Pearson’s correlation analysis demonstrated a significant positive relationship between the two morphometric parameters (*r* = 0.417, *p* < 0.001; *n* = 100 measurements from five sites; Fig. 4), indicating that increases in one structural variable were associated with increases in the other.

## Discussion

The skin of *L. petersii* exhibited the basic structure commonly reported in teleost fishes, in which the ED and DM are separated by BM, with skeletal muscle located beneath the dermal layer (Akat et al. 2022; Gabr et al. 2023). However, several distinct histological features were identified that may be associated with the ecological conditions and life-history characteristics of this species. These features include: (i) a DM characterized by a prominent SC with an almost complete absence of the stratum laxum, (ii) the presence of CCs within the ED, (iii) MCs predominantly distributed in the outermost epidermal layer, and (iv) regional variation in BT correlated with ET.

The DM of *L. petersii* showed an almost complete absence of the stratum laxum layer that is commonly reported in many teleost fishes (Madkour et al. 2023), whereas the SC was relatively well developed. In general, the stratum laxum of teleost skin consists of loosely arranged connective tissue containing collagen fibers, blood vessels, and fibroblasts (Abed et al. 2023). These collagenous structures contribute to maintaining tensile strength, buffering mechanical stress generated by external forces or water flow, and supporting the overall structural stability of the integument (Zhang et al. 2023). However, the dermal connective tissue of *L. petersii* showed a reduction in collagen-rich stratum laxum, strongly stained blue by Masson’s trichrome staining. Similar reductions or absence of dermal connective tissue layers have been reported in certain small-bodied or transparent fishes as well as in larval stages of many teleost fishes, where connective tissue development is often limited (Le Guellec et al. 2004). Therefore, the reduced development of the stratum laxum in *L. petersii* may be associated with maintaining an extremely thin and translucent body surface. Such a simplified dermal organization may also facilitate physiological exchange across the body surface and may reflect adaptive responses to environmental fluctuations encountered during the life cycle of this species (Rakers et al. 2010).

CCs identified within the ED are generally recognized as large secretory cells lacking direct openings to the external surface and are known to release biologically active substances associated with chemical alarm signaling and immune defense following tissue damage or environmental stress (Barreto et al. 2010). The appearance of CCs in *L. petersii* therefore implies that this species retains typical epidermal defense mechanisms capable of responding to environmental disturbances, despite possessing an extremely thin body surface.

MCs of *L. petersii*, distributed in the outermost epidermal layer, are responsible for secreting mucus that forms a protective mucosal barrier on the skin surface (Cabillon and Lazado 2019). Their mucus exhibited a purplish coloration in hematoxylin and eosin staining and a reddish-purple coloration in Masson’s trichrome staining. Such staining reactions are generally associated with secretions rich in mucopolysaccharides and glycoproteins (Bancroft and Gamble 2008). Fish epidermal mucus mainly consists of mucin-type glycoproteins, sulfated polysaccharides, and various bioactive substances, which together form a viscous protective layer on the skin surface (Shephard 1994). This mucus layer performs multiple functions, including reducing friction during swimming and providing protection against pathogens, parasites, and environmental stressors (Shephard 1994). Similar histochemical characteristics have been reported in several teleost fishes possessing abundant mucous cells, including certain gobies (Kim et al. 2019), the European eel *Anguilla anguilla* (Archer 1979), and various small benthic teleosts (Kim 2023). In these species, mucus rich in glycoproteins forms a protective mucosal barrier that acts as a first-line biochemical defense while maintaining hydration and lubrication of the epidermal surface (Ángeles Esteban 2012).

Because *L. petersii* possesses an extremely thin and translucent body surface, the mechanical protective function of the integument itself may be relatively limited. Under such conditions, mucus secreted by mucous cells, together with substances released from CCs, may serve as primary chemical and physiological defense mechanisms protecting the ED from environmental stressors. Such defense mechanisms may be particularly important in coastal and estuarine environments where fluctuations in salinity, suspended particles, and microbial loads frequently occur (Subramanian et al. 2008).

A significant positive correlation was observed between ET and BT in *L. petersii* (*r* = 0.417, *p* < 0.001). The concurrent thickening of the ED and BM may reinforce the structural connection between them, thereby enhancing tissue stability and contributing to the protective function of the integument by buffering mechanical impacts such as abrasion (Gu et al. 2023). Similar structural characteristics have been reported in benthic fishes inhabiting environments where frequent contact with substrates or friction caused by suspended particles occurs (Madkour et al. 2023). Therefore, the relatively thicker skin observed in the head and dorsal body regions of *L. petersii* may represent a histological strategy that protects body tissues from environmental stressors and may also serve as morphological evidence of adaptation to coastal and estuarine habitats. In surveyed skin locations, compared to other estuarial gobiid fishes (137.3 ± 8.5㎛ in *Tridentiger brevispinis*, 130 ~ 188㎛ in *Periophthalmus modestus*) (Park et al. 2000; Kim 2022), *Leucopsarion petersii* however exhibited a relatively thinner overall skin thickness (70.5 ± 28.6, *n* = 100), which is considered a histological adaptation that enables more rapid and efficient material exchange between the skin and the external environment in response to rapidly changing environmental conditions (Merkin et al. 2025).

Consequently, the skin structure of *L*. *petersii*, characterized by a simplified dermal organization, abundant MCs, the presence of CCs, and relatively thinner skin may reflect a morphological and histological adaptation that enables this species to cope with the fluctuating environmental conditions of coastal and estuarine habitats.

## Conclusion

This research investigated the histological and morphometric organization of the skin in the Korean ice goby *Leucopsarion petersii*, focusing on characteristics associated with its anadromous life history and adaptation to fluctuating coastal and estuarine environments. Microscopy revealed the following features of the skin: (i) a dermis characterized by a well-developed stratum compactum with an almost complete absence of the stratum laxum, (ii) the presence of club cells within the epidermis, (iii) mucous cells predominantly distributed in the outermost epidermal layer, and (iv) significant regional variation in epidermal and basement membrane thickness with a positive correlation between these parameters. These findings suggest that *L. petersii* exhibits a specialized cutaneous structure in which reduced dermal complexity is compensated by enhanced mucosal and cellular defense mechanisms, representing an adaptive strategy for survival in environmentally dynamic estuarine habitats.

## Data Availability

Not applicable.

## Abbreviations

ED: Epidermis
DM: Dermis
BC: Basal cell
BM: Basement membrane
BT: Basement membrane thickness
CC: Club cell
ET: Epithelial thickness
H&E: Hematoxylin and eosin
MC: Mucous cell
OFC: Outermost flattened cell
SC: Stratum compactum
SM: Skeletal muscle layer
